# Multidrug-Resistant *Acinetobacter baumannii* Genetic Characterization and Spread in Lithuania in 2014, 2016, and 2018

**DOI:** 10.3390/life11020151

**Published:** 2021-02-16

**Authors:** Tatjana Kirtikliene, Aistė Mierauskaitė, Ilona Razmienė, Nomeda Kuisiene

**Affiliations:** 1 Department of Microbiology and Biotechnology, Institute of Biosciences, Life Sciences Center, Vilnius University, LT-10257 Vilnius, Lithuania; nomeda.kuisiene@gf.vu.lt; 2 National Public Health Surveillance Laboratory, Clinical Testing Department, LT-10257 Vilnius, Lithuania; aiste.mierauskaite@nvspl.lt (A.M.); ilona.razmiene@nvspl.lt (I.R.)

**Keywords:** multidrug resistance, nosocomial infections, *Acinetobacter baumannii*, MLVA-8, resistance genes

## Abstract

Bacterial resistance to antimicrobial agents plays an important role in the treatment of bacterial infections in healthcare institutions. The spread of multidrug-resistant bacteria can occur during inter- and intra-hospital transmissions among patients and hospital personnel. For this reason, more studies must be conducted to understand how resistance occurs in bacteria and how it moves between hospitals by comparing data from different years and looking out for any patterns that might emerge. Multidrug-resistant (MDR) *Acinetobacter spp.* was studied at 14 healthcare institutions in Lithuania during 2014, 2016, and 2018 using samples from human bloodstream infections. In total, 194 isolates were collected and identified using MALDI-TOF and VITEK2 analyzers as *Acinetobacter baumannii* group bacteria. After that, the isolates were analyzed for the presence of different resistance genes (20 genes were analyzed) and characterized by using the Rep-PCR and MLVA (multiple-locus variable-number tandem repeat analysis) genotyping methods. The results of the study showed the relatedness of the different *Acinetobacter spp*. isolates and a possible circulation of resistance genes or profiles during the different years of the study. This study provides essential information, such as variability and diversity of resistance genes, genetic profiling, and clustering of isolates, to better understand the antimicrobial resistance patterns of *Acinetobacter spp*. These results can be used to strengthen the control of multidrug-resistant infections in healthcare institutions and to prevent potential outbreaks of this pathogen in the future.

## 1. Introduction

*Acinetobacter* spp. are recognized as important opportunistic pathogens worldwide. Their contribution to nosocomial infections has increased over the years, and many outbreaks in hospitals involving *Acinetobacter spp*. have been reported. The most dangerous property of *Acinetobacter* spp. is its ability to develop resistance to antibiotics extremely rapidly [1], and people who have weakened immune systems, chronic lung disease, or diabetes may be more susceptible to infections with *Acinetobacter*, especially patients with a prolonged hospital stay, those with open wounds, or any person with urinary catheters. *Acinetobacter* can be spread to susceptible persons through person-to-person contact or contact with contaminated surfaces.

*Acinetobacter* spp. have become resistant to almost all antimicrobial agents that are currently available, including aminoglycosides, quinolones, and broad-spectrum β-lactams. There are important differences in antimicrobial susceptibility between *A. baumannii* and other species in the genus furthermore, *A. baumannii* is known to be the most resistant species. All known mechanisms leading to antibiotic resistance, including enzymatic degradation, altered targets, and active efflux, have been found in *A. baumannii*. The genes associated with resistance can be chromosomal or located on plasmids. The mechanisms and epidemiology of carbapenem-resistant *A. baumannii* have been reviewed by Poirel and Nordmann [2].

Molecular characterization and strain typing of *Acinetobacter spp.* is important for the detection of the sources and model of infection spread, which is the main step in designing targeted infection control strategies. PCR and sequence-based methods are frequently used for genotyping *Acinetobacter spp.* and might be more appropriate for strain phylogeny and large-scale epidemiology [3]. Rep-PCR is simple, rapid, and has a high resolution for the identification and differentiation of *Acinetobacter spp*. isolates therefore, it has been invaluable for the epidemiological investigation of hospital outbreaks [4]. Multiple locus variable number of tandem repeats (VNTR) analysis (MLVA) schemes allow the identification of isolate distribution and the investigation of the population structure at a high resolution [4].

This study aimed to characterize multidrug-resistant *Acinetobacter spp.* isolates collected during 2014, 2016, and 2018 from different healthcare institutions in Lithuania and determine the transmission of resistance genes over the years in Lithuania.

## 2. Materials and Methods

### 2.1. Acinetobacter *spp.* Isolates Collection, Identification, and Genomic DNA Extraction

A total of 194 isolates of *Acinetobacter spp*. with resistance to β-lactams were selected from samples collected during 2014, 2016, and 2018 from 14 different healthcare institutions in Lithuania taken from patients with bloodstream infection. All the isolates were cultivated on blood agar for 17–24 h, and pure cultures were identified using a MALDI-TOF biotyper (Brucker). Antibiotic resistance to β-lactam antibiotics was confirmed by the disc diffusion method on Müller-Hinton agar and an automated VITEK-2 system (bioMérieux, France). Antibiotic resistance data were evaluated using EUCAST Acinetobacter spp. breakpoints guidance and commercial disks of antibiotics were used: Meropenem 10 µg, ciprofloxacin—5 µg, trimethoprim/sulfamethoxazole—1.25/23.75 µg, gentamicin—10 µg, amikacin—30 µg, and imipenem—10 µg (BioMaxima, Lublin, Poland). 

To extract the genomic DNA of Acinetobacter spp., the isolates were cultivated at 30 °C for 12 h on tryptic soy agar. Genomic DNA was extracted using the GeneJET Genomic DNA Purification Kit (Thermo Fisher Scientific, Vilnius, Lithuania) according to the supplier’s protocol.

### 2.2. Characterization of Acinetobacter spp. Antibiotic Resistance Genes

After the collection and identification of *Acinetobacter spp*. isolates, antibiotic resistance genes were detected. A total of 28 genes and gene groups were identified using gene primers according to previously published studies, which are presented in Table 1. Antibiotic resistance genes were identified using PCR. The PCR reaction contained 50 µL of the reaction mixture using the DreamTaq Green PCR Master Mix (2×) (Thermo Fisher Scientific, Vilnius, Lithuania), 0.5 μM of primer, and 1 µL of Acinetobacter spp. isolates genomic DNA. After PCR analysis, the amplification products were analyzed using 1% agarose gel electrophoresis.

### 2.3. Genotyping of Acinetobacter *spp.* Isolates

To detect the spread of β-lactam-resistant *Acinetobacter *spp. isolates in Lithuania, several genotyping methods were used. First, BOX-PCR genotyping was performed. After the analysis of the BOX-PCR results and determination of isolates that belonged to the same strains, (GTG)5-PCR was performed on the isolates from the same strain. Both methods were described by Domberk et al. [16], and the PCR reactions were performed in 50 μL of reaction mixture containing DreamTaq Green PCR Master Mix (2X) (Thermo Fisher Scientific), 1 μM of primer (BOX-PCR: BOXA1R- 5′-CTA CGG CAA GGC GAC GCT GAC G-3′; (GTG)5 -5′-GTGGTGGTGGTGGTG-3′), and 1 µL of bacterial DNA. The amplification products were analyzed using electrophoresis on a 1% agarose gel. 

After genotyping with BOX-PCR and (GTG)5—PCR, the MLVA method was performed as described by Pourcel et al. (2011). The PCR reaction mixture contained the DreamTaq Green PCR Master Mix (2×) (Thermo Fisher Scientific, Vilnius, Lithuania). The products of MLVA were analyzed via electrophoresis using 2% agarose gel for L repeats and 3% agarose gel for S repeats as described by Pourcel et al. [17]. The number of tandem repeats in each of the 8 loci was calculated manually based on the size of the PCR products. The size of the PCR products was the sum of the primers, tandem repeats, and the sequence between the primers and tandem repeats. The values of flanking region size referred to the genome of strain *A. baumannii* ATCC 17978. 

### 2.4. Data Analysis

Electrophoresis profiles of *Acinetobacter* spp. BOX-PCR and (GTG)5—PCR products were analyzed using BioNumerics 7.0 software (Applied Maths, Sint-Martens-Latem, Belgium). After the detection of the profiles, dendrograms were constructed using the unweighted-pair group method with the arithmetic mean (UPGMA) grouping method. 

The MLVA electrophoresis profiles were analyzed manually, and the variable number of tandem repeats was calculated as described by Pourcel et al. [17]. A dendrogram was constructed from the MLVA-8 results using the unweighted pair group method with the arithmetic mean (UPGMA) grouping method.

χ^2^ test or Fisher’s exact test was used for the comparison of categorical variables. A *P* value of <0.05 was considered to be statistically significant. Statistical analyses were performed using SPSS 10.0 software (SPSS Inc., Chicago, IL, USA).

## 3. Results

### 3.1. Acinetobacter *spp.* Identification, Phenotypic Characterization, and Detection of Antibiotic Resistance Genes

During the different years of study (2014, 2016, and 2018), 194 clinical isolates of *Acinetobacter* spp. were collected from 14 healthcare institutions in Lithuania. All the isolates were identified as *Acinetobacter baumannii* group bacteria. Phenotypic resistance identification according to EUCAST recommendations showed that all the isolates were resistant to the third-generation cephalosporins (one or more antibiotics) and/or carbapenems (one or more). After phenotypic resistance confirmation, genetic resistance was confirmed in all the identified isolates. For this reason, we divided the analyzed resistance genes into four groups: Genes responsible for aminoglycoside resistance, quinolone resistance, β-lactams resistance, and efflux pump regulators. Screening of these gene groups showed that the most common resistance genes were bla_OXA subgroup-3_ (100% of all isolates), bla_OXA-51_ (98.9%), bla_OXA subgroup-1_ (99.5%), bla_OXA subgroup-2_ (97%), bla_OXA subgroup-4_ (86%), bla_VIM-1_ (76.2%), bla_TEM-92_ (69.9%), bla_GES-11_ (66%), bla_parC_ (63%), bla_aaC2_ (61.3%), and bla_aphA6_ (52.1%). Eight of these gene groups (bla_OXA subgroup-3_, bla_OXA-51_, bla_OXA subgroup-1_, bla_OXA subgroup-2_, bla_OXA subgroup-4_, bla_VIM-1_, bla_TEM-92_, and bla_GES-11_) are responsible for resistance to β-lactams, two genes (bla_aacC2_ and bla_aphA6_) indicate resistance to aminoglycosides, and one gene, bla_parC_, showed resistance to quinolones. Resistance genes bla_adeE_ and bla_aacC1_ were not found in any isolates.

**Figure 1:**
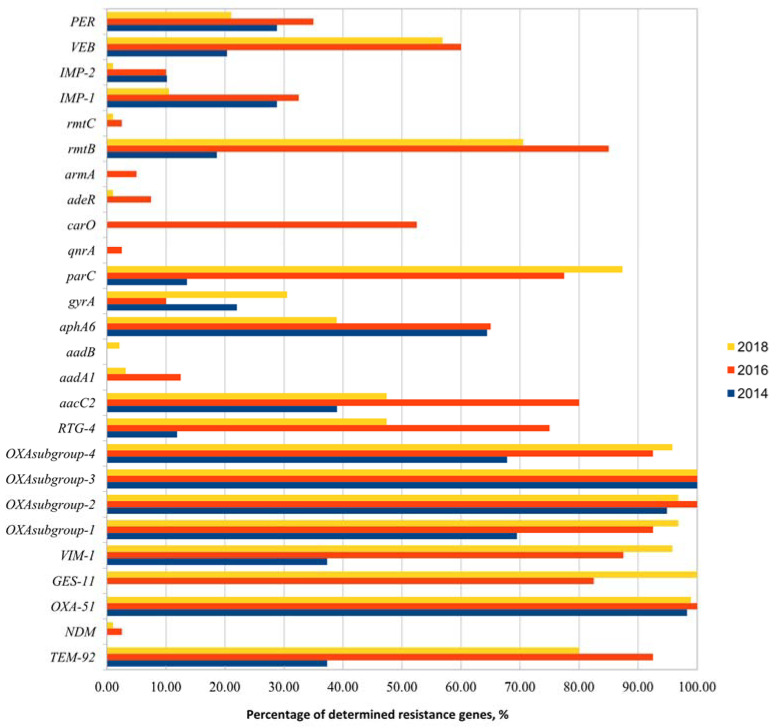
Analysis of detected resistance genes of *Acinetobacter spp*. during 2014, 2016, and 2018.

A comparison of isolates from the different years showed that the variety of the analyzed resistance genes differed between years differs. The highest variety of resistance genes was identified in isolates from 2018 and the lowest was from 2014. During each of these years of study, more diverse genes were identified year by year, but a few of the analyzed genes, which were found in isolates from 2016, were not present in isolates from 2018 (Figure 1). Further data analysis showed that the highest number of identified resistance genes belonged to isolates collected during 2016 and the highest number of resistance genes belonged to isolates 2016240 (number of genes = 17), 2016177 (n = 17), and 2016246 (n = 17). The lowest number of identified antibiotic resistance genes (10 genes or less) was observed in isolates collected in 2014. The lowest number of genes was found in isolates 201483 (number of genes = 4), 2014162 (n = 5), and 2014238 (n = 4). 

The composition of resistance genes changed between years. In comparison with 2014, in 2016, eight new genes were observed: bla_NDM_ (number of isolates from 2016 isolates = 1), bla_GES-11_ (n = 33), blaaadA1 (n = 5), bla_armA_ (n = 2), bla_rmtC_ (n = 1), bla_qnrA_ (n = 1), bla_carO_ (n = 21), and bla_adeR_ (n = 3). A comparison of 2018 isolates with 2016 showed one new resistance gene, which was not observed in isolates from previous years—bla_aadB_ (number of isolates = 2), but three genes were missed in comparison with 2016—bla_qnrA_, bla_carO_, and bla_armA_. 

### 3.2. Rep-PCR and MLVA Genotyping Results

BOX-PCR genotyping analysis was performed for all 194 characterized isolates. A total of 191 BOX-PCR profiles were identified, and a dendrogram was created (not presented), cluster 4 (n = 36, 18.9%), and cluster 2 (n = 33, 17.3%). The dominant cluster 6 had isolates mostly collected from 2014 (20 isolates) and 2018 (20 isolates). Only one cluster, cluster 3, had isolates from only one year of the collection (2018). The cluster comparison results are presented in Table 2. 

Three BOX-PCR profiles had two isolates each. The isolates were collected during 2014 (one profile) and 2018 (two profiles). All the isolates were collected from different healthcare institutions (A, C, G, and H). Further analysis with (GTG)_5_-PCR allowed us to determine that all these isolates were genetically very similar, the only exception being the isolate—2018444 (hospital H), which showed a genetic difference of 50% from all other compared isolates (Figure S1). Moreover, the detection of antibiotic resistance genes showed that the analyzed isolates had different resistance gene profiles, different gene combinations, and different gene numbers (Table 3). The lowest number of resistance genes showed isolates 201429 and 2014212 (7 and 8 genes), and another four isolates showed a higher number of genes: 2018364: 12 genes, 2018444: 11 genes, 2018391: 12 genes, and 2018419: 14 genes. Resistance gene combinations differed in each of the six isolates, mostly in the detection of aminoglycoside resistance genes and β-lactams (Table 3).

The occurrence of eight VNTR loci was assessed in 194 collected isolates. Two VNTR markers (Abaum_3530 and Abaum_3002) obtained the same product sizes in all selected isolates. Six VNTR markers (Abaum_1988, Abaum_2240, Abaum_2396, Abaum_3468, Abaum_0826, and Abaum_0845) showed polymorphism, and the variation in the number of repeats was calculated using visual calculations of the PCR products. The amplification products were obtained from all isolates with all VNTR markers. 

The 194 tested strains showed high genetic diversity as revealed via Rep-PCR. A total of 125 different MLVA profiles were detected in comparison with 191 BOX-PCR profiles (Figure S2). The dendrogram constructed using the UPGMA separated all the detected profiles in six clusters. An analysis of BOX-PCR and MLVA-8 clusters showed that these clusters have a variety of different resistance genes in all profiles, and moreover, the presence of genes was not restricted to one exact cluster. 

Using the MLVA profiles of *Acinetobacter spp*., it was discovered that more than six isolates (which were discovered by BOX-PCR) were of the same strain. Most isolates belonging to the same strain were from hospitals A, H, and M. Moreover, some isolates from the same strain had variations in resistance gene combinations and they either had or did not have some of the genes. In total, 125 strains of *Acinetobacter spp.* were detected by comparing the BOX-PCR and MLVA genotyping methods.

## 4. Discussion

The number of cases of antimicrobial-resistant bacterial nosocomial infections around the world has been growing over the past decade [18]. One of the highest rates of resistance in healthcare institutions is observed in *Acinetobacter spp.* isolates, which causes outbreaks around the world and is highly adaptable to changes in the environment and changes in the use of antibiotics in healthcare institutions. These characteristics lead to a high rate of occurrence of multidrug-resistant *Acinetobacter spp.* cases in the environment. Moreover, some studies have shown that over the years *Acinetobacter spp.* antimicrobial resistance is changing and the bacteria have become almost immune to all known treatments with antibiotics [19]. Our study aimed to evaluate the changes in *Acinetobacter baumannii* resistance in isolates collected from different years. Due to the high risks they pose to healthcare institutions, only the multidrug-resistant *Acinetobacter spp*. isolates were examined. During this study, 24 resistance genes were analyzed, which are responsible for resistance to three groups of antibiotics: Aminoglycosides, quinolones, and β-lactams, and one group of the gene, which is responsible for efflux pump regulation. An analysis of the aminoglycoside resistance genes detected showed that the most common resistance genes were: bla_rmtB_ (57.7% of all isolates), bla_aphA6_ (52.1%), and blaa_acC2_ (51.6%). The bla_rmtB_ gene is associated with 16S rRNA methylase production, which causes enzymatic modification at the methylation of 16S rRNA and makes *Acinetobacter spp*. isolates highly resistant to all clinically important aminoglycosides. In 2003, clinical isolates of highly aminoglycoside-resistant gram-negative bacteria producing16S rRNA methylase were identified in France [20], Japan [21], and other parts of the world, including Asian countries such as Afghanistan, Bangladesh, China, Hong Kong, India, Korea, Oman, and Pakistan [22]. Another resistance gene, encoding aminoglycoside—modifying enzyme phosphotransferase APH (3’)-Via is bla_aphA6_. bla_aacC2_ encodes another enzyme, acetyltransferase AAC (3)- IIa, which together with APH enzyme produces the highest levels of aminoglycoside resistance around the world [23]. Moreover, an evaluation of aminoglycoside resistance in our study showed that over different years, the number of different resistance genes grew, but only during the analysis of resistance genes blaa_acC2_, bla_aadA1_, bla_aphA6_, and bla_rmtB_ statistical significance was determined (*p* < 0.05). During 2014, isolates with bla_aacC2_, bla_aphA6_, and bla_rmtB_ genes were detected. During 2016, new genes were identified: bla_aadA1_, blaa_rmA_, and bla_rmtC_. Analysis of the data of *Acinetobacter baumannii* isolates collected in 2018 showed a new gene, bla_aadB_, but the bla_armA_ gene, which was detected during 2016, was absent. These data show the variability and diversity of resistance genes in *Acinetobacter spp*. isolates during the years under study.

Worldwide studies of aminoglycoside resistance have shown some differences in antibiotic resistance genes. Moniri et al. [24] evaluated aminoglycoside resistance genes of 60 *Acinetobacter* strains isolated from hospitalized patients in Iran and reported the presence of the bla_aacC1_ gene in 63.3% of *Acinetobacter* isolates, bla_aphA6_, bla_aadA1_, and bla_aadB_ genes were detected in 65%, 41.7%, and 3.3% of the isolates, respectively. In another study conducted by Nigro et al. [25] in Australia, a pattern of resistance to aminoglycosides was investigated in 61 multidrug-resistant *A. baumannii* isolates collected between 2000 and 2010 in six Australian hospitals. The isolates were screened for aminoglycoside modifying genes: bla_aadB_, bla_aacC1_, bla_aphA1b_, and bla_aphA6_ and it was found that the bla_aphA6_ gene was present in combination with bla_aacC1_ and bla_aphA1_ in a few isolates [24]. These results differ from the results of our study, where bla_accC1_ was not detected at all, and the bla_aadB_ gene was detected only in two of the isolates collected during 2018. 

Three quinolone resistance genes were observed during this study: bla_gyrA_, bla_parC_, and bla_qnrA_. bla_gyA_ (23.7% of isolates) and bla_parC_ (62.9% of isolates) were detected in all the years under study. Both genes are part of the most frequent mechanisms of resistance to quinolones, including alterations in genes that encode subunits of the quinolone target DNA gyrase (bla_gyrA_), and topoisomerase IV (bla_parC_) [26]. bla_qnrA_ was found in only one isolate, collected in 2016, which is contrary to other studies, where bla_qnrA_ is commonly found worldwide in Enterobacteriaceae bacteria [26,27] and *Acinetobacter baumannii* isolates [28]. For further studies, gene sequencing must be performed for the analysis of mutations appearing in the detected genes.

The highest number of antibiotic resistance genes, which were observed during this study, were genes responsible for resistance to β-lactams. Gene groups, that had the highest number of isolates: OXA subgroup-3 (found in all collected isolates; genes: bla_OXA-51_, bla_OXA-64_, bla_OXA-65_, bla_OXA-66_, bla_OXA-68_, bla_OXA-69_, bla_OXA-70_, bla_OXA-71_, bla_OXA-75_, bla_OXA-76_, bla_OXA-77_, and bla_OXA-78_), OXA subgroup-2 (bla_OXA-24,_ bla_OXA-25_, bla_OXA-26_, bla_OXA-40_, and bla_OXA-72_), which contains 96.9% of all isolates, and OXA subgroup-1 (bla_OXA-23_, bla_OXA-27_, and bla_OXA-49_) containing 87.6% of all isolates. In previous studies in Lithuania [29], several genes from these groups were detected: bla_OXA-24/40-like_ and bla_OXA-23-like_. Recently, it has been suggested that enzymes belonging to the OXA-51-like subgroup are intrinsic to *Acinetobacter baumannii* [30], occurring in most or all strains, although they are variably expressed, and these results correlate with the results of this study, where the OXA-51 gene was found in 99% of all isolates. The same results were obtained in Poland healthcare units, where bla_OXA-51_ was obtained in all investigated isolates (n = 125) [31].

Carbapenems are one of the most important β-lactam antibiotics that are used worldwide for multidrug-resistant *Acinetobacter baumannii* treatment. Carbapenem resistance caused by acquiring metallo-beta-lactamases (MBLs) is considered to be more serious than other resistance mechanisms because MBLs can hydrolyze almost all beta-lactam antibiotics. Furthermore, MBL-encoding genes located on integrons can be disseminated easily from one bacterium to another [32]. For the first time in Lithuania, metallo-β-lactamase coding genes were observed during this study: bla_IMP-1_, bla_IMP-2_, bla_VIM-1_, and bla_NDM_. The most common MBL coding genes were bla_VIM-1_ (76.3% of all isolates) and bla_IMP-1_ (20.6%). These results are comparable to those of previous studies in Iran [33] and differ from the results observed in Poland, where *A. baumannii* strains harbouring bla_VIM_ were very rare, detected only in two isolates [31] and no isolates had bla_IMP_.

The analysis of our results showed that the percentage of bacteria with bla_VIM-1_ resistance gene increased from 2014 (37.29%) to 2018 (95.79%), but the percentage of bacteria having bla_IMP-1_ and bla_IMP-2_ decreased from 2014 (28.31% and 10.53%) to 2018 (10.17% and 1.05%, respectively). One possible explanation for this change could be the replacement of genes for the effective survival of *Acinetobacter spp*. isolates because of changes in antibiotic treatment strategies. 

Multidrug resistance in *Acinetobacter spp*. isolates include not only target-modifying enzymes but also the overexpression of the outer membrane active efflux system. Compared to other resistance mechanisms, active efflux pumps are more widely distributed and have a wider substrate range, which results in resistance to a high range of antibiotics [34]. An important resistance determinant in *A. baumannii* is the AdeABC efflux pump, which can change the expression from inductive to constitutive overexpression. Overexpression is possible as a result of various mutations or inactivation by insertion sequences in the local regulatory genes [35,36]. The AdeABC operon possesses two genes that encode proteins that act as a sensor (bla_adeS_) and a regulator (bla_adeR_) of the pump. During this study, the bla_adeR_ gene was identified only in 2% of the *Acinetobacter spp.* isolates, which differs from previous studies in the USA [5], where 95% of all isolates had bla_adeR_ regulator. The overexpression of the AdeABC efflux pump must be evaluated in detail in further studies. 

Evaluation of relatedness in clinical isolates of *Acinetobacter spp*. is essential to establish the route of transmission of multidrug-resistant isolates, predict epidemics at the national level and worldwide, provide infection control knowledge for improving hygiene policy, and avoid intrahospital transmission of multidrug-resistant isolates [37]. Rep-PCR is a strain-specific group of genotyping methods, which has previously been shown to be a highly selective molecular typing method for *Acinetobacter spp*. isolates [38]. In this study, BOX-PCR and (GTG)_5_–PCR methods were used for the genotyping of *Acinetobacter* spp. clinical isolates in Lithuania. During the study, isolates with identical BOX-PCR electrophoretic profiles were identified (six isolates from hospitals A, C, G, and H) and identification was confirmed using (GTG)_5_–PCR, which showed the high relatedness of these isolates. Moreover, cluster analysis of the BOX-PCR dendrogram showed that the dominant clusters 6 (24%) and 4 (18.9%), represented the majority of the detected genetic profiles. Clusters 4, 1, and 6 had isolates, which were collected from at least eight different healthcare units. Moreover, all these isolates were collected in all three years of study. Endemic clusters that have reappeared over the years were also detected in clusters 2 and 5. Only one cluster (cluster 3) was isolated only in 2018. A possible explanation for these results could be the successful adaptation of profile clusters along the years and their dissemination, which could occur through transmission from medical staff, contaminated equipment, or patient transfer from one healthcare institution to another [39]. These data confirmed our previously described results that clinical isolates of *Acinetobacter *spp. are involved in the intra- and/or inter-hospital dissemination between Lithuanian healthcare institutions [40]. Transmission of endemic clusters of *Acinetobacter spp*. over the years has also been described worldwide [37] and confirms that cluster analysis requires the constant evaluation of dissemination at the national level and worldwide because their persistence indicates adaptation to the hospital environment and represents a risk of more dangerous future outbreaks. 

The multiple-locus variable-number tandem repeat analysis (MLVA) is highly repetitive when used for typing *A. baumannii* [41] and results are comparable with the results worldwide [42]. For this reason, MLVA is a promising method for its expected robustness and communication between laboratories, which will enable laboratories to identify *Acinetobacter* spp. strains with those of an internet-based library [43].

By comparing the results of this study and studies worldwide [42,44,45], it was found that all eight VNTR regions were detected in this study, but in other studies, some of the loci were not observed or observed only in a limited number of isolates and that data indicate a high degree of variability and polymorphism in some of the VNTR loci. Moreover, the poor amplification of some loci suggests that the distribution of VNTR loci differs between isolates from different geographical regions [44]. The highest variability and polymorphism in locus repeat numbers detected in this study belonged to Abaum_0845 (10 different repeat numbers) and Abaum_0826 (9 repeat numbers). These results can be explained by the high variability of S-repeats, as described previously [17]. After the analysis of the MLVA-8 dendrogram and the formed clusters, the same genetic trend was found in the BOX-PCR results. There was no correlation between the clustering of isolates and specific years of study, and the isolates that form one profile were collected from different years. Moreover, different resistance genes were presented in genetically similar profiles. MLVA–8 complements the Rep-PCR results showing the transmission of genetic profiles during the different years under study. For further confirmation of these results, additional sequencing analysis must be performed to identify the origin of transmission (chromosomal or plasmid-associated) and a number of recombination in different non-chromosomal genetic elements.

After comparing the data from different years under study, it was detected that the highest number of resistant genes were in isolates collected during 2016 and the lowest in 2014. According to the BOX-PCR and MLVA-8, the isolates of the different years were distributed proportionally in all detected clusters, except for isolates from 2018, which had the highest numbers from the beginning. Moreover, the study results showed that the variety of analyzed resistance genes differed from year to year and more antimicrobial resistance genes are detected each year. These data show that *Acinetobacter spp.* resistance mechanisms in Lithuania are changing. The results also showed that different genotyping profiles carried different resistance genes. Such differences between the results of genotyping and searching for resistance genes can be explained by the localization of some antimicrobial resistance determinants on different mobile elements [38] and needs further study. 

In conclusion, based on the rep-PCR and MLVA genotyping genetic profiles and clusters of related isolates were determined. A variety of resistance genes were detected. Furthermore, we showed that the distribution of genetic profiles changes year by year. It was observed in the genetic profiles of studied isolates that during the different years under study, some resistance profiles were transmitted with some changes in resistance gene combinations. However, further study is necessary to confirm that multidrug-resistant *A. baumannii* isolates adapt to the changing strategies of antibiotic use.

## Figures and Tables

**Table 1:** Antibiotic resistance genes characterized during the study.

Resistance Gene	Resistance Mechanism	Reference
*aacC1*	Amynoglycoside-modifying enzymes (AMEs)	[5]
*aacC2*		
*aadB*		
*aadA1*		
*aphA6*		
*rmtB*		[6]
*rmtC*		
*armA*		
*gyrA*	Fluoroquinolone resistance-determining regions (QRDR)	[5]
*parC*		
*qnrA*		
*adeR*	Efflux pumps regulation genes	[5]
*adeE*		
*blaPER-1*	β-lactams resistance genes	[7]
*blaVEB-1*		[8]
*blaGES-11*		[9]
*blaTEM-92*		[10]
*blaRTG-4*		[11]
*blaIMP-1*		[12]
*blaIMP-2*		
*blaVIM-1*		[13]
*blaNDM*		[14]
blaOXA subgroup-1 (*bla_OXA-23_*, *bla_OXA-27_*, *bla_OXA-49_*)		[8]
blaOXA subgroup-2 (*bla_OXA-24_*, *bla_OXA-25_*, *bla_OXA-26_*, *bla_OXA-40_*, *bla_OXA-72_*)		
blaOXA subgroup-3 (*bla_OXA-51_*, *bla_OXA-64_*, *bla_OXA-65_*, *bla_OXA-66_*, *bla_OXA-68_*, *bla_OXA-69_*, *bla_OXA-70_*, *bla_OXA-71_*, *bla_OXA-75_*, *bla_OXA-76_*, *bla_OXA-77_*, *bla_OXA-78_* )		
blaOXA subgroup-4 (*bla_OXA-58_*)		
*blaOXA-51*		[15]
*blacarO*		[5]

**Table 2:** BOX-PCR dendrogram clusters analysis.

Cluster	2014 Year: Number of Profiles	2016 Year: Number of Profiles	2018 Year: Number of Profiles	Number of Healthcare Institutions	Numbers of Profiles in Healthcare Institutions (Number of Profiles)
Cluster 1	14	1	1	9	A (11), D (1), E (1), F (1), G (1), H (8), K (1), M (1), N (1)
Cluster 2	5	1	27	8	A (12), C (2), D (3), F (1), G (2), H (8), I (1), K (2), one A/H profile, one A/G profile
Cluster 3	-	-	20	7	A (9), C (2), D (1), E (1), F (1), H (4), I (2)
Cluster 4	11	22	2	10	A (16), B (1), D (1), E (2), F (1), H (7), I (2), K (1), M (2), N (1)
Cluster 5	8	9	13	8	A (15), D (2), F (1), J (1), H (4), K (3), L (1), M (3)
Cluster 6	20	6	20	9	A (19), B (1), C (1), D (1), F (1), G (1), H (11), I (2), M (7), one A/C profile

**Table 3:** Data comparison of isolates analyzed using (GTG)_5_-PCR.

Profile Number	Isolate	Healthcare Institution	Number of Resistance Genes Detected	Genes in Both Isolates	Different Genes in Isolates
1	201429	A	7	bla*_OXA-51_*, bla_VIM-1_, bla_OXA subgroup-2_, bla*_OXA subgroup-3_*	bla_aacC2_, bla_aphA6_, bla_IMP-1_
	2014212	G	8		bla_TEM-92_, bla_OXA subgroup-1_, bla_aacC1,_ bla_armA_
2	2018364	A	12	bla_TEM-92_, bla_OXA-51_, bla_GES-11_, bla_VIM-1_, bla_OXA subgroups 1-4_, bla_parC_, bla_VEB_	bla_gyrA_, bla_PER_
	2018444	H	11		bla_aacC2_
3	2018391	C	12	bla_TEM-92_, bla_OXA-51,_ bla_GES-11_, bla_VIM-1_, bla_OXA subgroups 1-4_, bla_RTG-4_, bla_aacC2_, bla_parC_, bla_rmtB_	-
	2018419	A	14		bla_aphA6_, bla_PER_

## Data Availability

Not applicable.

## Supplementary Materials

The following are available online at https://www.mdpi.com/2075-1729/11/2/151/s1, Figure S1: *Acinetobacter baumanii* (GTG)_5_-PCR analysis results. Figure S2: Dendrogram of MLVA-8 and resistance genes profiles from isolated *Acinetobacter* spp. samples.
