# Supplementary material for: Multidrug-Resistant Acinetobacter baumannii Genetic Characterization and Spread in Lithuania in 2014, 2016, and 2018
Source: Life (Basel). 2021 Feb 16;11(2):151. doi: 10.3390/life11020151 (PMC7920459; doi:10.3390/life11020151)
Supplement: Supplementary file 1 [file life-11-00151-s001.zip › Supplementary materials_final/Figure S2. _original.pdf]

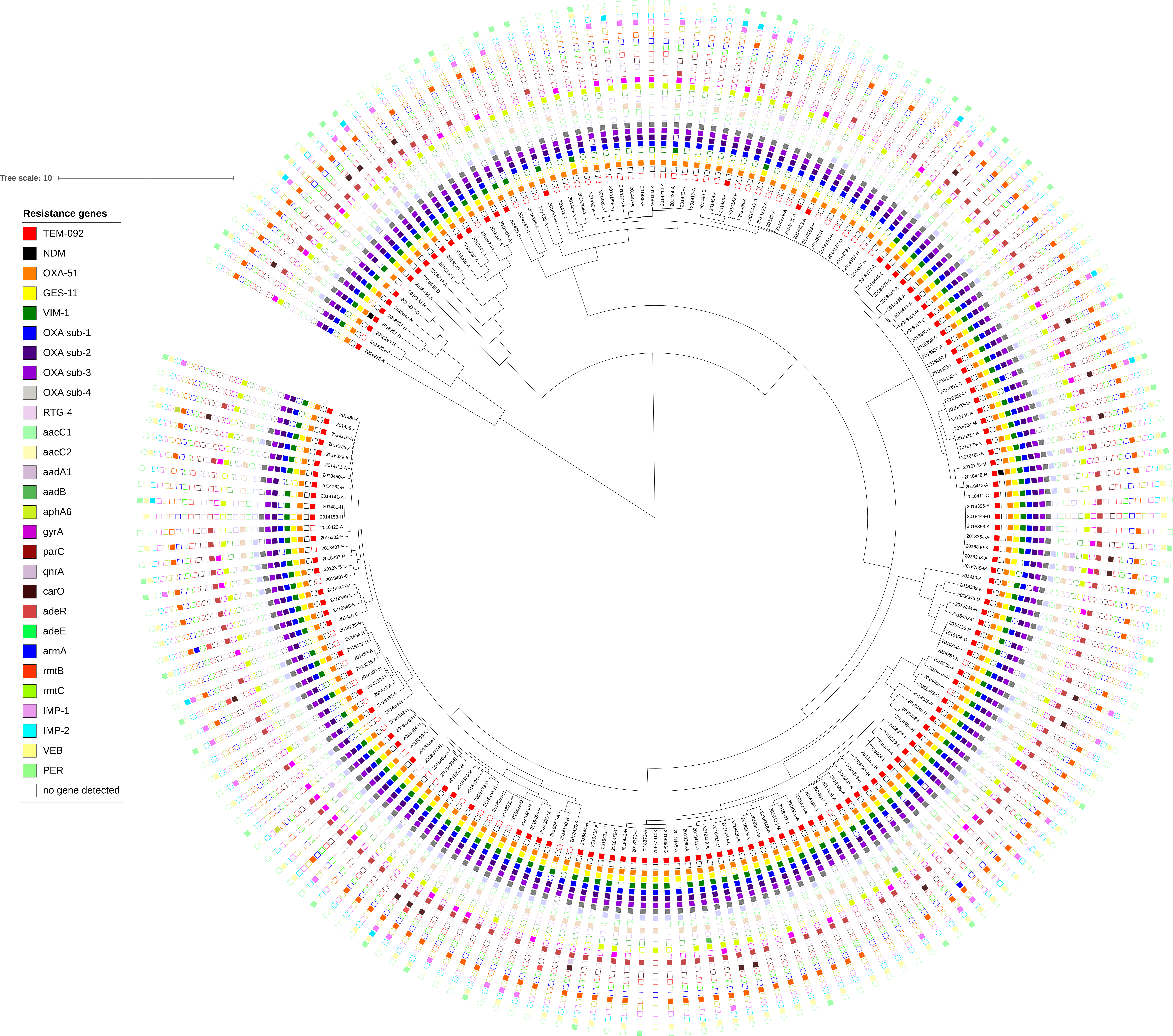

Tree scale: 10
Resistance genes
TEM-092
NDM
OXA-51
GES-11
VIM-1
OXA sub-1
OXA sub-2
OXA sub-3
OXA sub-4
RTG-4
aacC1
aacC2
aadA1
aadB
aphA6
gyrA
parC
qnrA
carO
adeR
adeE
armA
rmtB
rmtC
IMP-1
IMP-2
VEB
PER
no gene detected
