# Supplementary figures and images for: Multidrug-Resistant Acinetobacter baumannii Genetic Characterization and Spread in Lithuania in 2014, 2016, and 2018
Source: Life (Basel). 2021 Feb 16;11(2):151. doi: 10.3390/life11020151 (PMC7920459; doi:10.3390/life11020151)

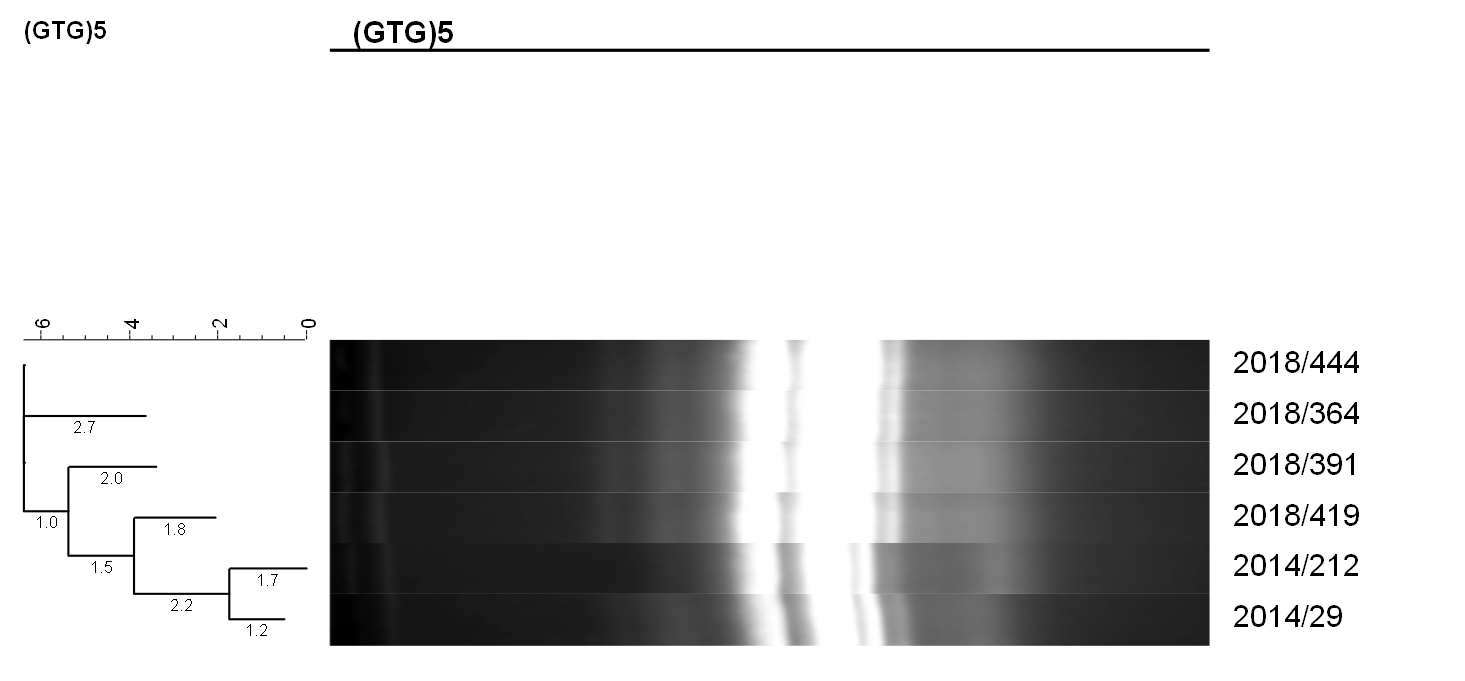

Supplement: Supplementary file 1 [file life-11-00151-s001.zip › Supplementary materials_final/Figure S1._original.tiff]
